# Supplementary material for: Content-rich biological network constructed by mining PubMed abstracts
Source: BMC Bioinformatics. 2004 Oct 8;5:147. doi: 10.1186/1471-2105-5-147 (PMC528731; doi:10.1186/1471-2105-5-147)
Supplement: Additional File 2 — The original results of the above study (non-essential files are deleted to keep the file size under the limit set by BMC bioinformatics). [file 1471-2105-5-147-S2.bz2 › chilibotAdditionalFile2/dip50/294ID9309223E1126/html/NRDA_NRDB.html]

 


 **NRDA** and **NRDB** 
  
Found 1800 abstracts in PubMed, retrieved 50.  
 

 What does Google say? 
 PDF only 
| .edu only 

---

**Interactive relationship** (e.g. stimulation, inhibition, etc)

**Neutral relationship**- Wild type p53 regulates human ribonucleotide reductase  [ **NRDB** ]   [ **NRDA** ]  by protein protein interaction with p53R2 as well as hRRM2 subunits.  Ref: 12615712 Cancer Res, 2003

**Non-interactive relationship** (e.g. studied together, co-existance, homology, etc.)

- GTI 2040, an Antisense Agent Targeting the Small Subunit Component R2 of Human Ribonucleotide Reductase  [ **NRDB** ]   [ **NRDA** ] , Shows Potent Antitumor Activity against a Variety of Tumors.  Ref: 12782585 Cancer Res, 2003
- However, cytotoxicity may also result from inhibition of DNA synthesis, due to either 1 dFdCyd diphosphate mediated inhibition of ribonucleotide reductase  [ **NRDB** ]   [ **NRDA** ] , or 2 direct inhibition of DNA polymerases by the 5 triphosphate of dFdCyd dFdCTP.  Ref: 12811514 Cancer Chemother Pharmacol< MedlineTA>Cancer Chemother Pharmacol, 2003
- MGI114, ET743, BBR3464, ZD0473, ZD9331, BN80915, J107088, F11782, XR11576, BMS247550, PS341, UCN01, ISIS 3521, STI571, ZD1839, IMC C225, OSI774, SU5416, DNA minor and major grooves, chimeric proteins, ribonucleotide reductase  [ **NRDB** ]   [ **NRDA** ] , topoisomerases, tubuline, proteasome, protein kinase C, bcr abl, EGF or VEGF tyrosine kinase receptors are code names somewhat barbarian and targets for new drugs which will complement the therapeutic arsenal of the twenty first century oncologist.  Ref: 12609801 Bull Cancer, 2003
- An assay of nucleotide permeable cells for ribonucleotide reductase  [ **NRDB** ]   [ **NRDA** ]  activity using 3H CDP as substrate revealed a pre early and complete decline of DNA precursor biosynthesis not found in the untreated control.  Ref: 12882290 Biotechnol Lett, 2003
- In addition, poplar glutaredoxin is able to reduce the Escherichia coli ribonucleotide reductase  [ **NRDB** ]   [ **NRDA** ]  1a in vitro reduction of cytidine diphosphate.  Ref: 12626113 Antioxid Redox Signal, 2003
- Ribonucleotide reductase  [ **NRDB** ]   [ **NRDA** ]  RR, the rate limiting enzyme of DNA synthesis, seems to be a complementary target for combination chemotherapy of brain tumors.  Ref: 12690517 Cancer Chemother Pharmacol< MedlineTA>Cancer Chemother Pharmacol, 2003
- Ribonucleotide reductase  [ **NRDB** ]   [ **NRDA** ]  RNR catalyzes the essential production of deoxyribonucleotides in all living cells.  Ref: 12562785 J Bacteriol, 2003
- The p53R2 gene encodes the p53 inducible ribonucleotide reductase  [ **NRDB** ]   [ **NRDA** ]  small subunit 2 homologue, which is part of the p53 pathway.  Ref: 12565178 Cancer Lett, 2003
- The R2 protein of class I ribonucleotide reductase  [ **NRDB** ]   [ **NRDA** ]  generates and stores a tyrosyl radical essential for ribonucleotide reduction and, thus, DNA synthesis.  Ref: 12624184 Proc Natl Acad Sci U S A, 2003
- Survival of DNA damage in yeast directly depends on increased dNTP levels allowed by relaxed feedback inhibition of ribonucleotide reductase  [ **NRDB** ]   [ **NRDA** ] .  Ref: 12581528 Cell, 2003
- The stumpy nature of pCPTcAMP treated parasites was confirmed by 1 morphological change, 2 inhibition of growth and DNA synthesis, 3 cell cycle arrest in the G 1 G 0 phase, 4 expression of NADH diaphorase activity and dihydrolipoamide dehydrogenase, 5 disappearance of the small subunit of ribonucleotide reductase  [ **NRDB** ]   [ **NRDA** ] , 6 up regulation of the major lysosomal membrane protein, and 7 efficient transformation into replicating procyclic insect forms after induction with citrate cis aconitate.  Ref: 12594963 Exp Parasitol, 2002
- Structure of the Large Subunit of Class Ib Ribonucleotide Reductase  [ **NRDB** ]   [ **NRDA** ]  from Salmonella typhimurium and its Complexes with Allosteric Effectors.  Ref: 12818204 J Mol Biol, 2003
- Neither the timing nor extent of gene induction was inhibited when factor arrested dbf4 cells were released into medium containing the ribonucleotide reductase  [ **NRDB** ]   [ **NRDA** ]  inhibitor hydroxyurea, which blocks replication fork progression, or were released at 37 degrees C, which blocks replication origin firing.  Ref: 12643263 J Biol Chem, 2003
- Hydroxyurea inhibits ribonucleotide reductase  [ **NRDB** ]   [ **NRDA** ]  and increases the levels of fetal hemoglobin, which explains a portion of the effects of this drug.  Ref: 12570692 Curr Med Chem, 2003
- A number of other reactions of hydroxyurea including the reaction with ribonucleotide reductase  [ **NRDB** ]   [ **NRDA** ]  and irradiation demonstrate the potential to release nitric oxide and should be further investigated.  Ref: 12570692 Curr Med Chem, 2003
- From the similarity in iron center structure, we propose that the & ; mgr ; oxo bridge in oxidized desaturase is bound to the di iron center as in rubrerythrin and not as reported for the R2 subunit of ribonucleotide reductase  [ **NRDB** ]   [ **NRDA** ]  and the hydroxylase subunit of methane mono oxygenase.  Ref: 12704186 J Biol Chem< MedlineTA>J Biol Chem, 2003
- This increase is conferred by an unusual, relaxed dATP feedback inhibition of ribonucleotide reductase  [ **NRDB** ]   [ **NRDA** ]  RNR.  Ref: 12581528 Cell, 2003
- Mouse ribonucleotide reductase  [ **NRDB** ]   [ **NRDA** ]  R2 protein a new target for anaphase promoting complex Cdh1 mediated proteolysis.  Ref: 12655059 Proc Natl Acad Sci U S A, 2003
- Visualization of proliferating cells in the adult mammalian brain with the aid of ribonucleotide reductase  [ **NRDB** ]   [ **NRDA** ] .  Ref: 12834878 Brain Res, 2003
- First, after immunoprecipitation of MHC class I from NXS2 cells, peptides were eluted and examined in tandem MS analysis which lead to the identification of three novel natural MHC class I peptide ligands, TEALPVKLI from ribonucleotide reductase  [ **NRDB** ]   [ **NRDA** ]  M2, NEYIMSLI from Ser Thr protein phosphatase 2A and FEMVSTLI with unknown origin.  Ref: 12880984 Cancer Lett, 2003
- To elucidate the role of DNA synthesis inhibition in the cytotoxicity of dFdCyd, we compared dFdCyd to hydroxyurea HU, a ribonucleotide reductase  [ **NRDB** ]   [ **NRDA** ]  inhibitor, and aphidicolin, an inhibitor of DNA polymerases, in the U251 and D54 human glioblastoma cell lines.  Ref: 12811514 Cancer Chemother Pharmacol< MedlineTA>Cancer Chemother Pharmacol, 2003
- In these cells, two important enzymes in deoxynucleoside triphosphate production, ribonucleotide reductase  [ **NRDB** ]   [ **NRDA** ]  and thymidine kinase TK, both cell cycle regulated, are indeed very weakly expressed.  Ref: 12566084 Biochem Pharmacol, 2003
- Therefore, ribonucleotide reductase  [ **NRDB** ]   [ **NRDA** ]  activity is under exquisite control both transcriptionally and posttranscriptionally.  Ref: 12655059 Proc Natl Acad Sci U S A, 2003
- Sequences downstream of the transcription initiation site are important for proper initiation and regulation of mouse ribonucleotide reductase  [ **NRDB** ]   [ **NRDA** ]  R2 gene transcription.  Ref: 12694192 Eur J Biochem, 2003
- Statistically significant evidence of horizontal transfer was found in the case of six protein families DNA ligase, ribonucleotide reductase 1  [ **NRDB** ]   [ **NRDA** ] , SNF2 global transactivator, inhibitor of apoptosis, chitinase, and UDP glucosyltransferase.  Ref: 12716988 Mol Biol Evol, 2003
- Ribonucleotide reductase  [ **NRDB** ]   [ **NRDA** ]  RR plays a key role in the synthesis of DNA and is the only enzyme responsible for the reduction of ribonucleotides to their corresponding deoxyribonucleotides, providing a balanced supply of precursors for DNA synthesis and repair.  Ref: 12615712 Cancer Res, 2003
- The most interesting result of analysis of B12 elements is that B12 independent isozymes of the methionine synthase and ribonucleotide reductase  [ **NRDB** ]   [ **NRDA** ]  are regulated by B12 elements in bacteria that have both B12 dependent and B12 independent isozymes.  Ref: 12869542 J Biol ChemJ Biol Chem, 2003
- The NO induced dopaminergic cell loss causing Parkinson s disease PD has been postulated to include the following an inhibition of cytochrome oxidase, ribonucleotide reductase  [ **NRDB** ]   [ **NRDA** ] , mitochondrial complexes I, II, and IV in the respiratory chain, superoxide dismutase, glyceraldehyde 3 phosphate dehydrogenase.  Ref: 12880486 Antioxid Redox Signal, 2003
- Herein, we report the design, synthesis, and biological effects of nucleosides bearing a disulfide function on the sugar ring as prodrugs of potentially active mercaptonucleotides that can target ribonucleotide reductase  [ **NRDB** ]   [ **NRDA** ]  or reverse transcriptase.  Ref: 12801219 J Med Chem< MedlineTA>J Med Chem, 2003
- A Zn Cys 4 center has been found in the C terminal region of the crystal structure of the anaerobic class III ribonucleotide reductase  [ **NRDB** ]   [ **NRDA** ]  RNR from bacteriophage T4.  Ref: 12655046 Proc Natl Acad Sci U S A, 2003
- A metal binding site in the catalytic subunit of anaerobic ribonucleotide reductase  [ **NRDB** ]   [ **NRDA** ] .  Ref: 12655046 Proc Natl Acad Sci U S A, 2003
- The small subunit of Escherichia coli ribonucleotide reductase  [ **NRDB** ]   [ **NRDA** ]  R2 is a homodimeric betabeta protein, in which each beta peptide contains a diiron cluster composed of two inequivalent iron sites.  Ref: 12862469 J Am Chem Soc, 2003
- The nrdE gene product R1E, the large subunit of the class 1b Salmonella typhimurium ribonucleotide reductase  [ **NRDB** ]   [ **NRDA** ] , has been overexpressed, purified and crystallized.  Ref: 12777781 Acta Crystallogr D Biol Crystallogr, 2003
- In fission yeast, the COP9 signalosome is required to activate ribonucleotide reductase  [ **NRDB** ]   [ **NRDA** ]  for DNA synthesis.  Ref: 12867051 Curr Biol, 2003
- Comprehensive model for allosteric regulation of mammalian ribonucleotide reductase  [ **NRDB** ]   [ **NRDA** ]  refinements and consequences.  Ref: 12578384 Biochemistry< MedlineTA>Biochemistry, 2003
- One alternative approach is to use the enzyme ribonucleotide reductase  [ **NRDB** ]   [ **NRDA** ]  RNR, expressed by proliferating cells, but not by quiescent ones.  Ref: 12834878 Brain Res, 2003
- Theoretical Studies on the Mechanism of Inhibition of Ribonucleotide Reductase  [ **NRDB** ]   [ **NRDA** ]  by E 2 Fluoromethylene 2 deoxycitidine 5 diphosphate.  Ref: 12785865 J Am Chem Soc, 2003
- Short term treatment with novel ribonucleotide reductase  [ **NRDB** ]   [ **NRDA** ]  inhibitors Trimidox and Didox reverses late stage murine retrovirus induced lymphoproliferative disease with less bone marrow toxicity than hydroxyurea.  Ref: 12630679 Antivir Chem Chemother, 2002
- Two carboxylates and a single oxygen species have long been implicated as core elements of the elusive intermediate X in ribonucleotide reductase  [ **NRDB** ]   [ **NRDA** ] .  Ref: 12691585 Inorg Chem, 2003
- Expression and preliminary crystallographic studies of R1E, the large subunit of ribonucleotide reductase  [ **NRDB** ]   [ **NRDA** ]  from Salmonella typhimurium.  Ref: 12777781 Acta Crystallogr D Biol Crystallogr, 2003
- The probable mechanism is inhibition of both ribonucleotide reductase  [ **NRDB** ]   [ **NRDA** ]  and a protein tyrosine phosphatase.  Ref: 12776257 Semin Oncol, 2003
- Furthermore, overexpression of suc22, a ribonucleotide reductase  [ **NRDB** ]   [ **NRDA** ]  gene, fully complemented the sporulation defect of cdt2 mutants and alleviated their growth defect at 19 degrees.  Ref: 12871901 Genetics, 2003
- Ribonucleotide reductase  [ **NRDB** ]   [ **NRDA** ]  immunoreactivity in adenocarcinoma cells and malignant or reactive mesothelial cells in serous effusions.  Ref: 12685191 Acta Cytol< MedlineTA>Acta Cytol, 2003
- Deoxyribonucleoside 2 or 3 Mixed Disulfides Prodrugs To Target Ribonucleotide Reductase  [ **NRDB** ]   [ **NRDA** ]  and or To Inhibit HIV Reverse Transcription.  Ref: 12801219 J Med Chem< MedlineTA>J Med Chem, 2003
- Subcellular localization of yeast ribonucleotide reductase  [ **NRDB** ]   [ **NRDA** ]  regulated by the DNA replication and damage checkpoint pathways.  Ref: 12732713 Proc Natl Acad Sci U S A, 2003
- Expression of ferritin light chain, rhoA, inosine monophosphatdgehydrogenase II, ribonucleotide reductase  [ **NRDB** ]   [ **NRDA** ]  M2, pro2000 and pro1859 were increased after drug treatment.  Ref: 12740912 Int J Cancer, 2003
- E 2 Fluoromethylene 2 deoxycitidine 5 diphosphate FMCDP is a potent time dependent inactivator of the enzyme Ribonucleotide Reductase  [ **NRDB** ]   [ **NRDA** ] , which operates by destructing an essential tyrosil radical and performing a covalent addition to an active site residue.  Ref: 12785865 J Am Chem Soc, 2003
- In vivo assay for low activity mutant forms of Escherichia coli ribonucleotide reductase  [ **NRDB** ]   [ **NRDA** ] .  Ref: 12562785 J Bacteriol, 2003
- Most of these examples describe mechanisms where dioxygen is either formed, as in photosystem II, or cleaved as in many other enzymes like cytochrome c oxidase, ribonucleotide reductase  [ **NRDB** ]   [ **NRDA** ] , methane mono oxygenase and tyrosinase.  Ref: 12643044 Q Rev Biophys, 2003
- Displacement of the tyrosyl radical cofactor in ribonucleotide reductase  [ **NRDB** ]   [ **NRDA** ]  obtained by single crystal high field EPR and 1.4 A x ray data.  Ref: 12624184 Proc Natl Acad Sci U S A, 2003
- GTI 2040 is a 20 mer oligonucleotide that is complementary to a coding region in the mRNA of the R2 small subunit component of human ribonucleotide reductase  [ **NRDB** ]   [ **NRDA** ] .  Ref: 12782585 Cancer Res, 2003
- ribonucleotide reductase  [ **NRDB** ]   [ **NRDA** ]  intermediate X.  Ref: 12859213 Acc Chem Res, 2003
- Corynebacterium ammoniagenes class Ib ribonucleotide reductase  [ **NRDB** ]   [ **NRDA** ]  transcriptional regulation of an atypical genomic organization in the nrd cluster.  Ref: 12686643 Microbiology, 2003
- HSV 2 R1, the R1 subunit of herpes simplex virus HSV ribonucleotide reductase  [ **NRDB** ]   [ **NRDA** ] , protects cells against apoptosis.  Ref: 12804778 FEBS Lett, 2003
- Furthermore, plasmoredoxin provides electrons for ribonucleotide reductase  [ **NRDB** ]   [ **NRDA** ] , the enzyme catalyzing the first step of DNA synthesis.  Ref: 12631266 Eur J Biochem, 2003
- The three dimensional structure of the large subunit of the first member of a class Ib ribonucleotide reductase  [ **NRDB** ]   [ **NRDA** ] , R1E of Salmonella typhimurium, has been determined in its native form and together with three allosteric effectors.  Ref: 12818204 J Mol Biol, 2003
- RT PCR showed that all five genes are expressed late in infection compared to the early ribonucleotide reductase  [ **NRDB** ]   [ **NRDA** ]  large subunit gene.  Ref: 12771421 J Gen Virol, 2003
- Hydroxyurea HU, a ribonucleotide reductase  [ **NRDB** ]   [ **NRDA** ]  inhibitor, induces morphological anomalies in the central nervous system CNS, craniofacial tissues and limb buds in animals, and neonatal respiratory distress in humans.  Ref: 12647788 Histol Histopathol, 2003
- This is mediated via the ubiquitin ligase Pcu4, activation of which leads to degradation of the scaffold protein Spd1, which anchors the small ribonucleotide reductase  [ **NRDB** ]   [ **NRDA** ]  subunit in the nucleus away from the large subunit in the cytoplasm.  Ref: 12867051 Curr Biol, 2003
- Cop9 signalosome subunits and Pcu4 regulate ribonucleotide reductase  [ **NRDB** ]   [ **NRDA** ]  by both checkpoint dependent and independent mechanisms.  Ref: 12695334 Genes Dev, 2003
- The R1 subunit of herpes simplex virus ribonucleotide reductase  [ **NRDB** ]   [ **NRDA** ]  has chaperone like activity similar to Hsp27.  Ref: 12804778 FEBS Lett, 2003
- Ribonucleotide reductase  [ **NRDB** ]   [ **NRDA** ]  is essential for the synthesis of all four dNTPs required for DNA replication.  Ref: 12694192 Eur J Biochem, 2003
- p53R2, which is regulated by tumor suppressor p53, is a small subunit of ribonucleotide reductase  [ **NRDB** ]   [ **NRDA** ] .  Ref: 12858174 Nat GenetNat Genet, 2003
- We have previously shown that HQ and catechol inhibit ribonucleotide reductase  [ **NRDB** ]   [ **NRDA** ] , the rate limiting enzyme in DNA synthesis.  Ref: 12749906 Mol Immunol, 2003
- A density functional evaluation of an Fe III Fe IV model diiron cluster comparisons with ribonucleotide reductase  [ **NRDB** ]   [ **NRDA** ]  intermediate X.  Ref: 12691585 Inorg Chem, 2003
- Ribonucleotide reductase  [ **NRDB** ]   [ **NRDA** ]  consists of two nonidentical proteins, R1 and R2, and catalyzes the rate limiting step in DNA precursor synthesis the reduction of ribonucleotides to deoxyribonucleotides.  Ref: 12655059 Proc Natl Acad Sci U S A, 2003
- A simple model is presented to discuss the metabolic regulation of the new process based on the presence of a manganese ribonucleotide reductase  [ **NRDB** ]   [ **NRDA** ]  Mn RNR in the producing strain.  Ref: 12882290 Biotechnol Lett, 2003
- Hydroxyurea, a known S phase specific cytotoxic ribonucleotide reductase  [ **NRDB** ]   [ **NRDA** ]  RR inhibitor, is an effective agent for HbF induction in patients with SCA, but the mechanisms by which hydroxyurea induces HbF in vivo have not been elucidated.Materials and Methods.  Ref: 12842703 Exp Hematol< MedlineTA>Exp Hematol, 2003
- Mechanistic Implications for the Formation of the Diiron Cluster in Ribonucleotide Reductase  [ **NRDB** ]   [ **NRDA** ]  Provided by Quantitative EPR Spectroscopy.  Ref: 12862469 J Am Chem Soc, 2003
- Combined effects of temozolomide and the ribonucleotide reductase  [ **NRDB** ]   [ **NRDA** ]  inhibitors didox and trimidox in malignant brain tumor cells.  Ref: 12690517 Cancer Chemother Pharmacol< MedlineTA>Cancer Chemother Pharmacol, 2003
- Reduction of NDPs by murine ribonucleotide reductase  [ **NRDB** ]   [ **NRDA** ]  mRR requires catalytic mR1 and free radical containing mR2 subunits and is regulated by nucleoside triphosphate allosteric effectors.  Ref: 12578384 Biochemistry< MedlineTA>Biochemistry, 2003
- We evaluated the ability of a short course of treatment with the ribonucleotide reductase  [ **NRDB** ]   [ **NRDA** ]  RR inhibitor hydroxyurea HU and two novel RR inhibitors Trimidox TX and Didox DX to influence late stage murine retrovirus induced lymphoproliferative disease.  Ref: 12630679 Antivir Chem Chemother, 2002
- Ribonucleotide reductase  [ **NRDB** ]   [ **NRDA** ]  RNR catalyzes the rate limiting step in dNTP production and thus plays an essential role in DNA synthesis.  Ref: 12732713 Proc Natl Acad Sci U S A, 2003
- We find that two signalosome subunits, Csn1 and Csn2, are required to regulate ribonucleotide reductase  [ **NRDB** ]   [ **NRDA** ]  RNR through the degradation of a small protein, Spd1, that acts to anchor the small RNR subunit in the nucleus.  Ref: 12695334 Genes Dev, 2003
- Arrest of cell cycle by inhibition of ribonucleotide reductase  [ **NRDB** ]   [ **NRDA** ]  induces accumulation of NAD by Mn2 supplemented growth of Corynebacterium ammoniagenes.  Ref: 12882290 Biotechnol Lett, 2003
- In this issue of Cell, report on new aspects of the regulation of yeast ribonucleotide reductase  [ **NRDB** ]   [ **NRDA** ] , the mechanism by which dNTP levels are increased following DNA damage, and the consequences of the metabolic changes.  Ref: 12581518 Cell, 2003
- OBJECTIVE Ribonucleotide reductase  [ **NRDB** ]   [ **NRDA** ]  RNR is a cytoplasmic enzyme that is essential for DNA synthesis.  Ref: 12685191 Acta Cytol< MedlineTA>Acta Cytol, 2003
